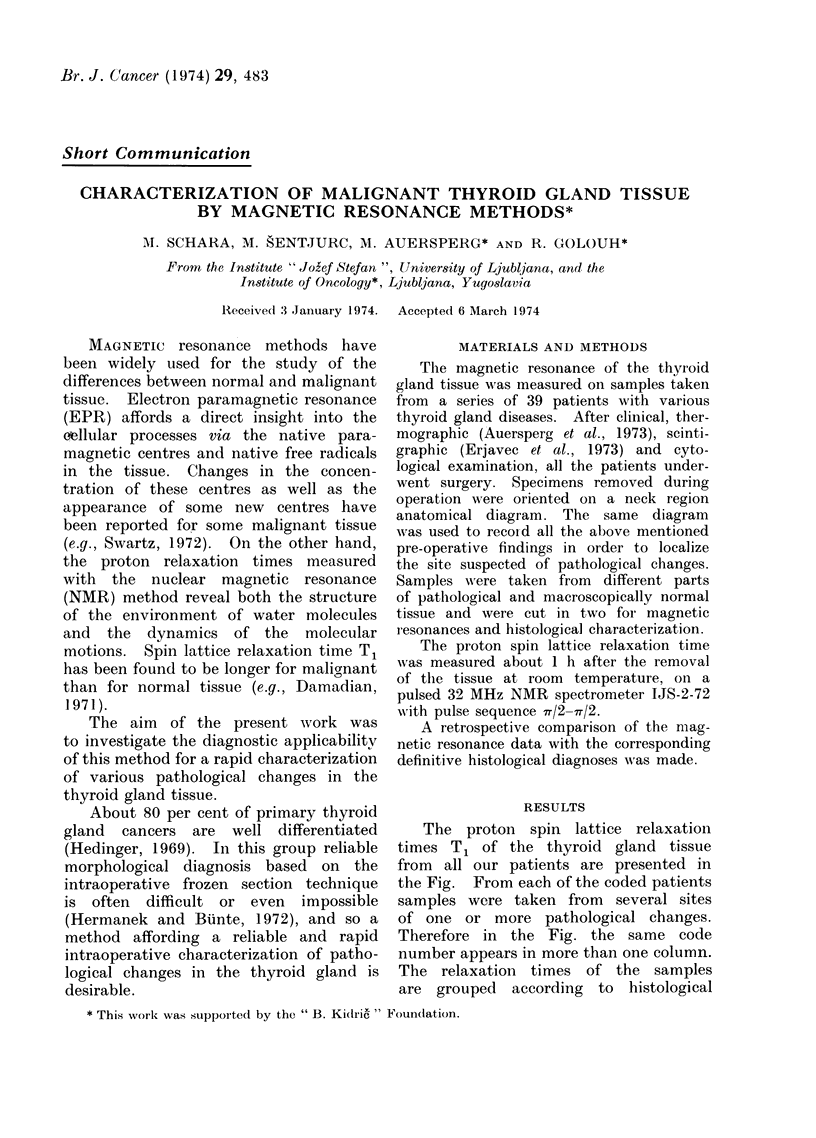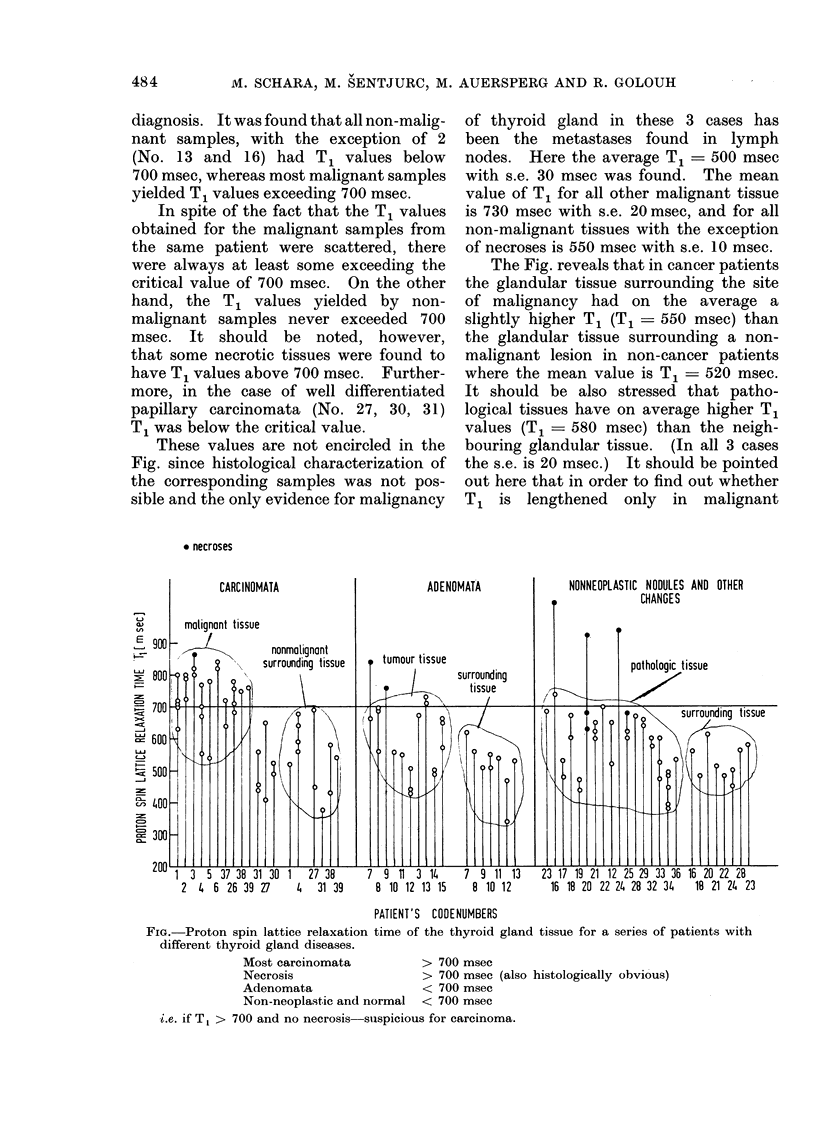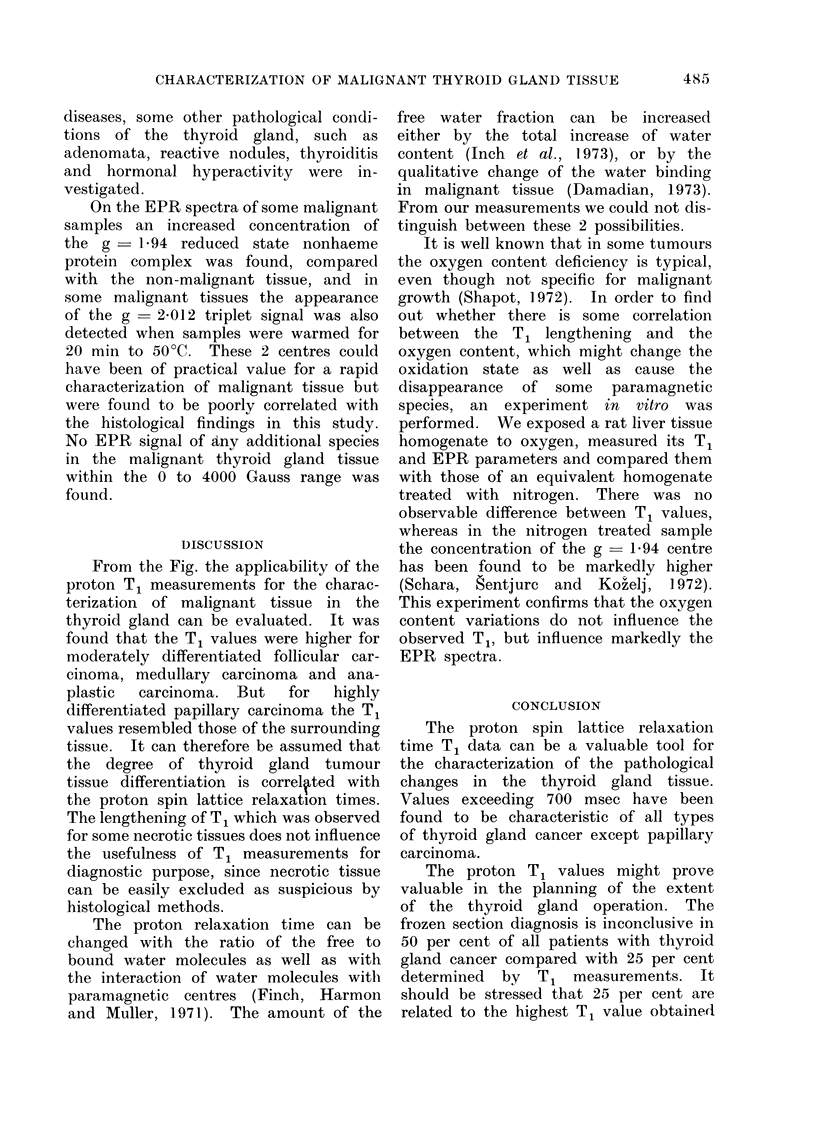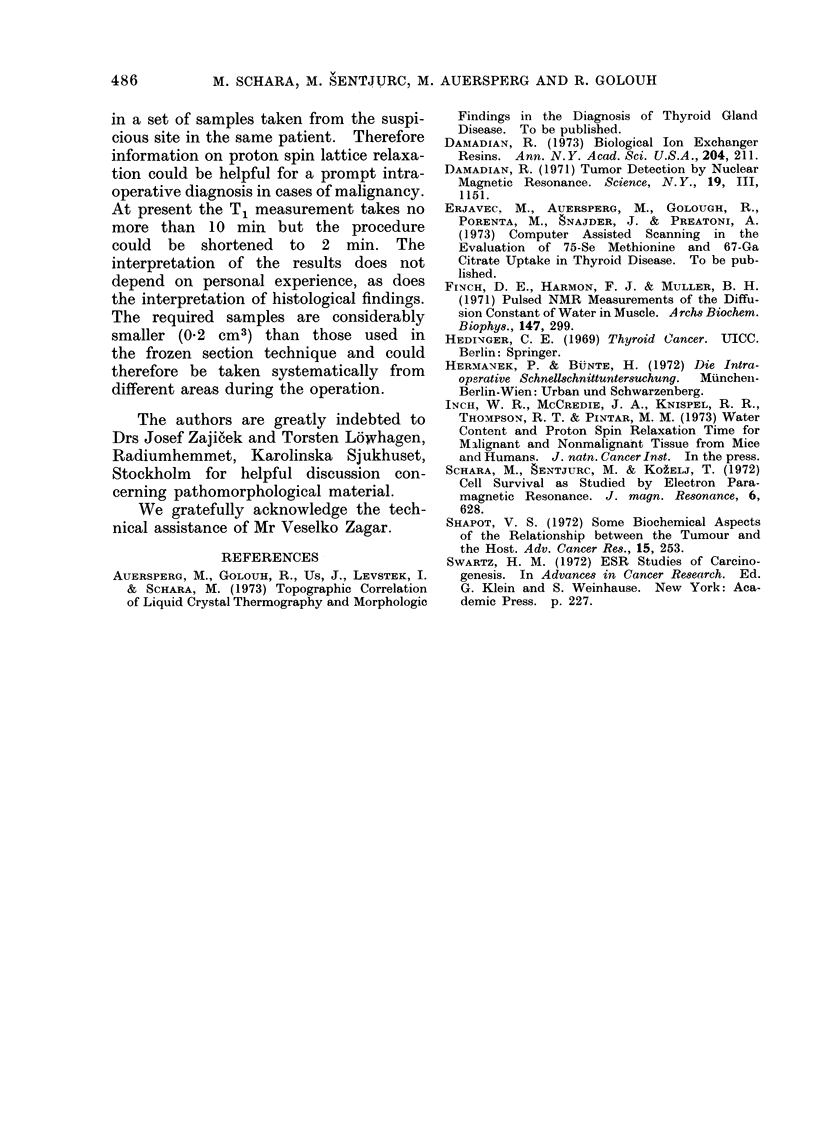# Characterization of malignant thyroid gland tissue by magnetic resonance methods.

**DOI:** 10.1038/bjc.1974.101

**Published:** 1974-06

**Authors:** M. Schara, M. Sentjurc, M. Auersperg, R. Golouh


*Br. J. Cancer* (1974) **29**, 483

### *Short Communication*

### CHARACTERIZATION OF MALIGNANT THYROID GLAND TISSUE BY MAGNETIC RESONANCE METHODS*

M. SCHARA, M. ŠENTJURC, M. AUERSPERG* AND R. GOLOUH*

*From the Institute "Jožef Stefan", University of Ljubljana, and the Institute of Oncology*, Ljubljana, Yugoslavia*

Received 3 January 1974. Accepted 6 March 1974

MAGNETIC resonance methods have been widely used for the study of the differences between normal and malignant tissue. Electron paramagnetic resonance (EPR) affords a direct insight into the cellular processes *via* the native paramagnetic centres and native free radicals in the tissue. Changes in the concentration of these centres as well as the appearance of some new centres have been reported for some malignant tissue (*e.g.*, Swartz, 1972). On the other hand, the proton relaxation times measured with the nuclear magnetic resonance (NMR) method reveal both the structure of the environment of water molecules and the dynamics of the molecular motions. Spin lattice relaxation time $T_1$ has been found to be longer for malignant than for normal tissue (*e.g.*, Damadian, 1971).

The aim of the present work was to investigate the diagnostic applicability of this method for a rapid characterization of various pathological changes in the thyroid gland tissue.

About 80 per cent of primary thyroid gland cancers are well differentiated (Hedinger, 1969). In this group reliable morphological diagnosis based on the intraoperative frozen section technique is often difficult or even impossible (Hermanek and Bünte, 1972), and so a method affording a reliable and rapid intraoperative characterization of pathological changes in the thyroid gland is desirable.

### MATERIALS AND METHODS

The magnetic resonance of the thyroid gland tissue was measured on samples taken from a series of 39 patients with various thyroid gland diseases. After clinical, thermographic (Auersperg *et al.*, 1973), scintigraphic (Erjavec *et al.*, 1973) and cytological examination, all the patients underwent surgery. Specimens removed during operation were oriented on a neck region anatomical diagram. The same diagram was used to record all the above mentioned pre-operative findings in order to localize the site suspected of pathological changes. Samples were taken from different parts of pathological and macroscopically normal tissue and were cut in two for magnetic resonances and histological characterization.

The proton spin lattice relaxation time was measured about 1 h after the removal of the tissue at room temperature, on a pulsed 32 MHz NMR spectrometer IJS-2-72 with pulse sequence $\pi/2-\pi/2$.

A retrospective comparison of the magnetic resonance data with the corresponding definitive histological diagnoses was made.

### RESULTS

The proton spin lattice relaxation times $T_1$ of the thyroid gland tissue from all our patients are presented in the Fig. From each of the coded patients samples were taken from several sites of one or more pathological changes. Therefore in the Fig. the same code number appears in more than one column. The relaxation times of the samples are grouped according to histological

* This work was supported by the "B. Kidrič" Foundation.

diagnosis. It was found that all non-malignant samples, with the exception of 2 (No. 13 and 16) had $T_1$ values below 700 msec, whereas most malignant samples yielded $T_1$ values exceeding 700 msec.

In spite of the fact that the $T_1$ values obtained for the malignant samples from the same patient were scattered, there were always at least some exceeding the critical value of 700 msec. On the other hand, the $T_1$ values yielded by non-malignant samples never exceeded 700 msec. It should be noted, however, that some necrotic tissues were found to have $T_1$ values above 700 msec. Furthermore, in the case of well differentiated papillary carcinomata (No. 27, 30, 31) $T_1$ was below the critical value.

These values are not encircled in the Fig. since histological characterization of the corresponding samples was not possible and the only evidence for malignancy of thyroid gland in these 3 cases has been the metastases found in lymph nodes. Here the average $T_1 = 500$ msec with s.e. 30 msec was found. The mean value of $T_1$ for all other malignant tissue is 730 msec with s.e. 20 msec, and for all non-malignant tissues with the exception of necroses is 550 msec with s.e. 10 msec.

The Fig. reveals that in cancer patients the glandular tissue surrounding the site of malignancy had on the average a slightly higher $T_1$ ($T_1 = 550$ msec) than the glandular tissue surrounding a non-malignant lesion in non-cancer patients where the mean value is $T_1 = 520$ msec. It should be also stressed that pathological tissues have on average higher $T_1$ values ($T_1 = 580$ msec) than the neighbouring glandular tissue. (In all 3 cases the s.e. is 20 msec.) It should be pointed out here that in order to find out whether $T_1$ is lengthened only in malignant

FIG.—Proton spin lattice relaxation time of the thyroid gland tissue for a series of patients with different thyroid gland diseases.

| | |
|---|---|
| Most carcinomata | > 700 msec |
| Necrosis | > 700 msec (also histologically obvious) |
| Adenomata | < 700 msec |
| Non-neoplastic and normal | < 700 msec |

*i.e.* if $T_1 > 700$ and no necrosis—suspicious for carcinoma.

diseases, some other pathological conditions of the thyroid gland, such as adenomata, reactive nodules, thyroiditis and hormonal hyperactivity were investigated.

On the EPR spectra of some malignant samples an increased concentration of the g = 1·94 reduced state nonhaeme protein complex was found, compared with the non-malignant tissue, and in some malignant tissues the appearance of the g = 2·012 triplet signal was also detected when samples were warmed for 20 min to 50°C. These 2 centres could have been of practical value for a rapid characterization of malignant tissue but were found to be poorly correlated with the histological findings in this study. No EPR signal of any additional species in the malignant thyroid gland tissue within the 0 to 4000 Gauss range was found.

### DISCUSSION

From the Fig. the applicability of the proton $T_1$ measurements for the characterization of malignant tissue in the thyroid gland can be evaluated. It was found that the $T_1$ values were higher for moderately differentiated follicular carcinoma, medullary carcinoma and anaplastic carcinoma. But for highly differentiated papillary carcinoma the $T_1$ values resembled those of the surrounding tissue. It can therefore be assumed that the degree of thyroid gland tumour tissue differentiation is correlated with the proton spin lattice relaxation times. The lengthening of $T_1$ which was observed for some necrotic tissues does not influence the usefulness of $T_1$ measurements for diagnostic purpose, since necrotic tissue can be easily excluded as suspicious by histological methods.

The proton relaxation time can be changed with the ratio of the free to bound water molecules as well as with the interaction of water molecules with paramagnetic centres (Finch, Harmon and Muller, 1971). The amount of the free water fraction can be increased either by the total increase of water content (Inch *et al.*, 1973), or by the qualitative change of the water binding in malignant tissue (Damadian, 1973). From our measurements we could not distinguish between these 2 possibilities.

It is well known that in some tumours the oxygen content deficiency is typical, even though not specific for malignant growth (Shapot, 1972). In order to find out whether there is some correlation between the $T_1$ lengthening and the oxygen content, which might change the oxidation state as well as cause the disappearance of some paramagnetic species, an experiment *in vitro* was performed. We exposed a rat liver tissue homogenate to oxygen, measured its $T_1$ and EPR parameters and compared them with those of an equivalent homogenate treated with nitrogen. There was no observable difference between $T_1$ values, whereas in the nitrogen treated sample the concentration of the g = 1·94 centre has been found to be markedly higher (Schara, Šentjurc and Koželj, 1972). This experiment confirms that the oxygen content variations do not influence the observed $T_1$, but influence markedly the EPR spectra.

### CONCLUSION

The proton spin lattice relaxation time $T_1$ data can be a valuable tool for the characterization of the pathological changes in the thyroid gland tissue. Values exceeding 700 msec have been found to be characteristic of all types of thyroid gland cancer except papillary carcinoma.

The proton $T_1$ values might prove valuable in the planning of the extent of the thyroid gland operation. The frozen section diagnosis is inconclusive in 50 per cent of all patients with thyroid gland cancer compared with 25 per cent determined by $T_1$ measurements. It should be stressed that 25 per cent are related to the highest $T_1$ value obtained in a set of samples taken from the suspicious site in the same patient. Therefore information on proton spin lattice relaxation could be helpful for a prompt intraoperative diagnosis in cases of malignancy. At present the $T_1$ measurement takes no more than 10 min but the procedure could be shortened to 2 min. The interpretation of the results does not depend on personal experience, as does the interpretation of histological findings. The required samples are considerably smaller (0·2 $cm^3$) than those used in the frozen section technique and could therefore be taken systematically from different areas during the operation.

The authors are greatly indebted to Drs Josef Zajiček and Torsten Löwhagen, Radiumhemmet, Karolinska Sjukhuset, Stockholm for helpful discussion concerning pathomorphological material.

We gratefully acknowledge the technical assistance of Mr Veselko Zagar.

### REFERENCES

Auersperg, M., Golouh, R., Us, J., Levstek, I. & Schara, M. (1973) Topographic Correlation of Liquid Crystal Thermography and Morphologic Findings in the Diagnosis of Thyroid Gland Disease. To be published.

Damadian, R. (1973) Biological Ion Exchanger Resins. *Ann. N.Y. Acad. Sci. U.S.A.*, **204**, 211.

Damadian, R. (1971) Tumor Detection by Nuclear Magnetic Resonance. *Science, N.Y.*, **19**, III, 1151.

Erjavec, M., Auersperg, M., Golough, R., Porenta, M., Šnajder, J. & Preatoni, A. (1973) Computer Assisted Scanning in the Evaluation of 75-Se Methionine and 67-Ga Citrate Uptake in Thyroid Disease. To be published.

Finch, D. E., Harmon, F. J. & Muller, B. H. (1971) Pulsed NMR Measurements of the Diffusion Constant of Water in Muscle. *Archs Biochem. Biophys.*, **147**, 299.

Hedinger, C. E. (1969) *Thyroid Cancer*. UICC. Berlin: Springer.

Hermanek, P. & Bünte, H. (1972) *Die Intraoperative Schnellschnittuntersuchung*. München-Berlin-Wien: Urban und Schwarzenberg.

Inch, W. R., McCredie, J. A., Knispel, R. R., Thompson, R. T. & Pintar, M. M. (1973) Water Content and Proton Spin Relaxation Time for Malignant and Nonmalignant Tissue from Mice and Humans. *J. natn. Cancer Inst.* In the press.

Schara, M., Šentjurc, M. & Koželj, T. (1972) Cell Survival as Studied by Electron Paramagnetic Resonance. *J. magn. Resonance*, **6**, 628.

Shapot, V. S. (1972) Some Biochemical Aspects of the Relationship between the Tumour and the Host. *Adv. Cancer Res.*, **15**, 253.

Swartz, H. M. (1972) ESR Studies of Carcinogenesis. In *Advances in Cancer Research*. Ed. G. Klein and S. Weinhause. New York: Academic Press. p. 227.